# Glucose metabolism across COVID-19 and long COVID: a longitudinal study

**DOI:** 10.1210/jendso/bvag173

**Published:** 2026-08-12

**Authors:** Joviane Daher, Ziad Koberssy, Mireya Diaz, Sokratis N Zisis, Clifford J Rosen, Grace A McComsey

**Affiliations:** School of Medicine, Case Western Reserve University, Cleveland, OH 44106, USA; School of Medicine, Case Western Reserve University, Cleveland, OH 44106, USA; Department of Population and Quantitative Health Sciences, School of Medicine, Case Western Reserve University, Cleveland, OH 44106, USA; Metrowest Medical Center, Tufts School of Medicine, Framingham, MA 01702, USA; Maine Medical Center Research Institute, Maine Medical Center, Scarborough, ME 04074, USA; School of Medicine, Case Western Reserve University, Cleveland, OH 44106, USA; Clinical Research Center, University Hospitals, Cleveland Medical Center, Cleveland, OH 44106, USA

**Keywords:** long COVID, COVID-19, hemoglobin A1c (HbA1c), inflammation, cardiometabolic disease

## Abstract

**Background:**

COVID-19 has been associated with new-onset diabetes. Prior studies are limited by lack of pre-infection data and preexisting cardiometabolic factors. Clarifying how COVID-19 and long COVID (LC) affect glucose metabolism is essential to guide prevention. We evaluated longitudinal changes in glycemic and cardiometabolic markers after SARS-CoV-2 infection in a prospective cohort with pre-infection baseline measurements.

**Methods:**

We included 821 uninfected adult participants from the RECOVER cohort who subsequently developed COVID-19, characterizing participants in their pre-COVID state. Data from up to 12 months post-infection were compared to pre-infection baseline. Participants with preexisting diabetes or pregnancy were excluded. The primary endpoint was change in HbA1c up to 12 months after infection. Analysis was stratified by LC status using the 2024 RECOVER Adult Long COVID Research Index (LCRI).

**Results:**

HbA1c remained stable from pre-infection to 12 months post-infection (5.39% vs 5.40%; *P* = .435). Non-HDL cholesterol and blood pressure decreased modestly, while cystatin C increased slightly; absolute changes were small. High-sensitivity C-reactive protein (hs-CRP), troponin, pro–brain natriuretic peptide (pro-BNP), body mass index, and waist circumference remained stable. The 10-year atherosclerotic cardiovascular disease (ASCVD) risk increased slightly (6.7% to 7.3%; *P* < .001). Trends were similar across LC groups. New-onset metabolic syndrome incidence was comparable across LC categories (18%-20%), although persistent metabolic syndrome was more frequent in participants with more severe LC.

**Conclusion:**

In this cohort with pre-infection baseline data, we found no significant changes in HbA1c or cardiometabolic biomarkers through 12 months after infection, although 10-year ASCVD risk slightly increased. These findings emphasize the importance of longitudinal assessment in differentiating transient physiological changes from persistent cardiometabolic disease after COVID-19.

COVID-19 is increasingly recognized as a systemic disease with multisystem involvement rather than a solely respiratory illness, as initially characterized (1, 2). Emerging evidence suggests an association between COVID-19 infection and adverse metabolic outcomes, including an increased risk of new-onset diabetes mellitus and worsening glycemic control (3-6). Proposed biological mechanisms include direct viral injury to pancreatic β-cells, systemic inflammation, hormonal changes, and immune dysregulation (7, 8). Together, these mechanisms raise concern that COVID-19 may have lasting effects on glucose metabolism beyond the acute phase of infection, rendering metabolic pathways vulnerable during and after infection. Recent studies reporting higher rates of diabetes following COVID-19 infection are limited by the lack of pre-infection glycemic data and by preexisting confounding factors such as obesity, prediabetes, vitamin D deficiency, and cardiometabolic disease, which may further confound observed metabolic changes (9, 10). Clarifying how COVID-19 affects glucose metabolism is therefore essential for primary prevention strategies.

Long COVID (LC), also referred to as post-acute sequelae of SARS-CoV-2 infection, is characterized by persistent symptoms and organ system involvement lasting weeks to months after the acute infection (11). Chronic inflammation, immune dysregulation, and autonomic and endocrine disturbances observed in LC provide a biologically plausible framework for sustained metabolic alterations, including impaired glucose regulation (12). However, data examining the relationship between LC and changes in glycemic control remain limited, and existing studies often lack standardized definitions of LC, longitudinal follow-up, or pre-infection metabolic measurements.

The RECOVER study provides a unique opportunity to address these gaps through its prospective design, enrollment of participants prior to SARS-CoV-2 infection, and longitudinal follow-up with standardized assessments (13). By capturing metabolic and clinical data before infection and following individuals through incident infection and recovery, RECOVER enables within-person comparisons that reduce confounding from baseline metabolic risk factors. In addition, the use of a standardized LC assessment allows for a more rigorous evaluation of the potential role of LC in post-infection metabolic changes.

The objective of this study was to evaluate changes in glycemic control following incident SARS-CoV-2 infection in adults without preexisting diabetes using pre-infection baseline measurements. The primary outcome was change in glycated hemoglobin (HbA1c) up to 12 months after infection compared with baseline. Secondary analyses examined whether glycemic trajectories differed by LC status, as well as assessed changes in cardiometabolic markers and 10-year atherosclerotic cardiovascular disease (ASCVD) risk up to 12 months after COVID infection.

## Methods

### Study design and population

We selected adult participants enrolled in the RECOVER study across 83 sites in 33 US states plus Washington DC, and Puerto Rico (13). Subjects could enter the study at different times in relation to their initial COVID infection or without a prior COVID infection, and they were defined into cohorts, including the acute infected (enrolled during first infection), post-acute (enrolled after initial COVID infection), and uninfected cohort (who enrolled prior to first infection but could be included in this study group if they were infected after study enrollment). For this study, we selected subjects enrolled without a known history of SARS-CoV-2 infection (uninfected) and had their first COVID-19 infection during follow-up period as defined by the World Health Organization (WHO) criteria (14). This design enabled us to characterize participants in their pre-COVID state. Individuals diagnosed with preexisting diabetes (HbA1c ≥ 6.5%) were excluded. Pregnant participants were excluded if the pregnancy period coincided with any of the data collection visits. Data from up to 12 months post-infection were compared to the pre-infection baseline. Pre-infection timepoint is defined as an “uninfected” participant's closest evaluation available from at least 7 days prior to their COVID-19 infection. Post-infection timepoint is defined as visits from 30 days and up to 395 days after the day of infection. Additionally, analysis was also stratified into 3 groups by LC status, assessed by the 2024 RECOVER Adult Long COVID Research Index (15) (LCRI) based on symptoms reported in surveys including: loss or change of smell and taste (+7 points on the score), post-exertional malaise (+6), chronic cough (+4), brain fogs (+3), thirst (+1), palpitations (+2), chest pain (+1), fatigue (+1), dizziness (+2), shortness of breath (+2), snoring or sleep apnea (+1). Subjects with an LCRI ≥ 11 were assigned to the LC group (LC+), and subjects with an LCRI of 1 to 10 were considered LC indeterminate (LCI), while those with an LCRI 0 were assigned to the LC negative group (LC−). Individuals were assigned to the LCRI group depending on the highest score observed in the post-infection period.

### Ethical considerations

Institutional review boards (IRB) at NYU Grossman School of Medicine and other participating institutions reviewed and approved the protocol. Further approval was obtained from the IRB at University Hospitals Cleveland Medical Center, Cleveland, Ohio. All participants were required to provide a written informed consent before participating in any research activity.

### Study measurements

#### Participant surveys

Participants completed every 90 days focused physical examination and surveys about baseline demographic information, medical history including COVID-19 infection history, concomitant medications, LC symptoms, and vaccination status (13).

#### Anthropometric, inflammatory, and cardiometabolic measures

Participants underwent physical and laboratory examinations at baseline “uninfected” visit(s), around infection (within 30 days of the infection), 6 months, and 12 months from infection. Participants underwent blood pressure measurements and anthropometric measurements, including waist circumference, weight, and height, from which body mass index (BMI) was calculated. Fasting was encouraged before each blood sample collection but was not mandated. These samples were sent to the local CLIA-certified laboratory for inflammatory and cardiometabolic measurements, including HbA1c, vitamin D (25(OH)D) levels, triglycerides (TG), total cholesterol, low-density lipoprotein (LDL), high-density lipoprotein (HDL), non-high-density lipoprotein (non-HDL), troponin, pro-B-type natriuretic peptide (pro-BNP), high-sensitivity C-reactive protein (hs-CRP), and cystatin C. We calculated the 10-year atherosclerotic cardiovascular disease (ASCVD) risk score before infection (ranging from days to months prior) and at 12 months post-infection using the American College of Cardiology ASCVD Risk Estimator Plus (16). We used the Joint Interim Statement of 2009 to define metabolic syndrome (17).

### Statistical analysis

Mean and standard deviation were used to characterize continuous variables, while counts and proportions were used for categorical variables. Unadjusted trajectories of cardiovascular and metabolic markers and their significance were examined via generalized estimating equations (GEE). Several correlation structures (eg, independence, exchangeable, autoregressive) among temporal observations were assessed and the one which led to the smallest Akaike Information Criterion was selected. Values of the markers from such model were estimated for 30 days prior to infection, the day of infection, 180 and 365 days after infection. The potential difference between LCRI groups was also assessed through the GEE model including corresponding terms for LCI and LC+ with the LC− as the reference group. Significance of changes for ASCVD before and after infection were assessed via paired *t* test within each LCRI group. The differences in distribution of different metabolic syndrome combinations across LCRI groups were assessed with the Fisher-Freeman-Halton test. Statistical significance was set at alpha = 0.05, unless there were multiple related tests in which case a Bonferroni adjustment was applied.

## Results

**Figure 1:**
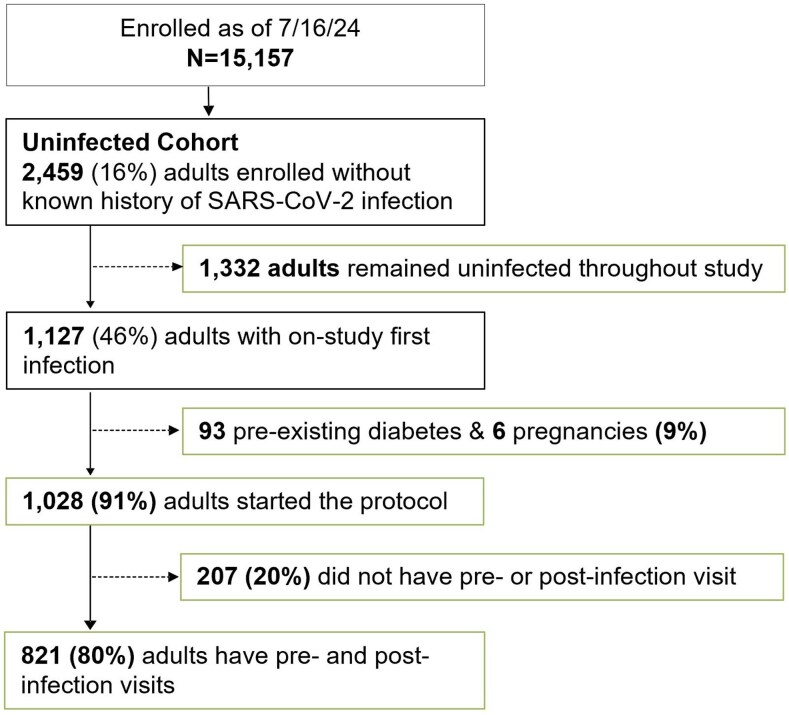
Strobe diagram of RECOVER incident infection cohort with pre- and post-infection visits. Of 15 157 adults enrolled, 1127 had the first SARS-CoV-2 infection during follow-up. After excluding preexisting diabetes (n = 93) and pregnancy (n = 6), 1028 initiated the protocol; 821 with both pre- and post-infection visits comprised the analytic cohort.

A total of 2459 participants were enrolled in the RECOVER uninfected cohort. 1127 participants had their first COVID-19 infection while on study. In total, 821 participants met our selection criteria and were included in this study. Figure 1 details the selection process of the cohort under analysis.

### Baseline characteristics

Among 821 participants with incident COVID-19 infection, LCRI was available for 778 individuals who were classified to the groups LC− (n = 342), LCI (n = 354), and LC+ (n = 82). Among the uninfected cohort, mean age was 51.4 years (±15.5 years) (Table 1). LC+ were younger with a mean age of 47.9 years (±15.6 years) compared to LC− (mean 52.2 ± 15.4 years) and LCI (mean 51.7 ± 15.5 years). Our cohort was predominantly of female sex at birth (75.5%), with a higher proportion of female individuals in the LC+ group (84.1%) compared to LC− (75.4%) and LCI (74.6%). Most participants identified as non-Hispanic White (72.3%), with similar racial and ethnic distributions across LC groups. Among the cohort, 87.3% were fully vaccinated at the time of infection. The proportion of fully vaccinated individuals was the lowest among LC+ participants (82.9%) compared with LC− (89.8%) and LCI (87.0%). Comorbid conditions and medications use were uncommon among participants. Only 2 subjects in the LCI group and 3 subjects in the total cohort were on metformin and glucagon-like peptide 1 receptor agonists (GLP1-RA), respectively.

**Table 1:** Descriptive of demographics and health status of COVID incident study cohort (n = 821) and by maximum LC status during follow-up at least 30 days after infection (n = 778)

Characteristic	Newly infected	LC−	LCI	LC+
N	821	342	354	82
Age at enrollment				
Mean (SD)	51.4 (15.5)	52.2 (15.4)	51.7 (15.5)	47.9 (15.6)
Sex assigned at birth				
Female	620 (75.5)	258 (75.4)	264 (74.6)	69 (84.1)
Male	196 (23.8)	82 (24.0)	90 (25.4)	12 (14.6)
Other/Unknown	5 (0.6)	2 (0.6)	0 (0)	1 (1.2)
Race & ethnicity				
NonH Asian	61 (7.4)	26 (7.6)	28 (7.9)	3 (3.7)
NonH Black	42 (5.2)	14 (4.1)	22 (6.2)	3 (3.7)
Hispanic	90 (11.1)	28 (8.2)	44 (12.4)	13 (15.9)
NonH White	593 (72.3)	262 (76.6)	249 (70.4)	57 (69.5)
Multiracial/Missing/Other	35 (4.3)	12 (3.5)	11 (3.1)	6 (7.3)
BMI at enrollment				
Mean (SD)	27.2 (6.0)	26.5 (5.1)	27.7 (6.7)	28.3 (5.9)
Vaccination status at index				
Unvaccinated	72 (8.8)	24 (7.0)	34 (9.6)	10 (12.2)
Partially vaccinated	9 (1.1)	3 (0.9)	5 (1.4)	1 (1.2)
Fully vaccinated	717 (87.3)	307 (89.8)	308 (87.0)	68 (82.9)
Date unknown/missing	23 (2.8)	8 (2.3)	7 (2.0)	3 (3.7)
Comorbidities at index				
Immunocompromised	29 (3.5)	4 (1.2)	16 (4.5)	7 (8.5)
Autoimmune	81 (9.9)	13 (3.8)	43 (12.1)	20 (24.4)
Cancer	27 (3.3)	6 (1.8)	19 (5.4)	2 (2.4)
Chronic liver disease	5 (0.6)	2 (0.6)	2 (0.6)	0 (0)
Kidney disease	7 (0.9)	2 (0.6)	5 (1.4)	0 (0)
CVD	40 (4.9)	13 (3.8)	17 (4.8)	8 (9.8)
Stroke	8 (1.0)	1 (0.3)	4 (1.1)	1 (1.2)
Asthma	113 (13.9)	30 (8.8)	60 (17.2)	16 (19.5)
COPD	8 (1.0)	1 (0.3)	3 (0.9)	4 (4.9)
Sickle cell anemia	2 (0.2)	0 (0)	1 (0.3)	0 (0)
Medications				
Statins	40 (4.9)	21 (6.1)	17 (4.8)	1 (1.2)
Steroids	3 (0.4)	1 (0.3)	1 (0.3)	1 (1.2)
Immunosuppressors	0 (0)	0 (0)	0 (0)	0 (0)
GLP-1 RAs	3 (0.4)	1 (0.3)	1 (0.3)	1 (1.2)
Metformin	2 (0.2)	0 (0)	2 (0.6)	0 (0)

Continuous values are presented as mean (SD).

Abbreviations: COPD, chronic obstructive pulmonary disease; CVD, cardiovascular diseases; GLP-1 RAs, glucagon-like peptide 1 receptor agonists; LC, long COVID; LCI, long COVID indeterminate; NonH, non-Hispanic.

### Changes in HbA1c and other cardiometabolic biomarkers

Unadjusted trajectories of cardiometabolic and inflammatory markers before and after SARS-CoV-2 infection are shown in Table 2. Most markers remained stable over the 1-year follow-up period. Mean HbA1c levels were unchanged over time (5.39% at index infection and 5.4% at 365 days; *P* = .435). Despite no significant longitudinal change in mean HbA1c over the follow-up period, 81 out of 821 participants met criteria for prediabetes during post-infection follow-up (defined as HbA1c 5.7%-6.4%) and 4 participants developed diabetes (HbA1c ≥ 6.5%) during this period. However, non-HDL cholesterol demonstrated a modest decline from 129.3 mg/dL at index infection to 125.8 mg/dL after a year (*P* < .001), whereas cystatin C showed a small but statistically significant increase from 0.87 mg/L at index infection to 0.91 mg/L at 365 days (*P* < .001). Blood pressure also decreased modestly during follow-up: mean systolic blood pressure declined from 123.4 mmHg at −30 days before infection to 122.4 mmHg at 365 days (*P* < .001), while mean diastolic blood pressure decreased from 76.6 mmHg to 76.3 mmHg over the same period (*P* = .04). Other biomarkers, including troponin-I, pro-BNP, D-dimer, hs-CRP, vitamin D, BMI, and waist circumference did not demonstrate significant changes during follow-up.

**Table 2:** Trajectories of cardiovascular/metabolic markers before and after (up to 1 year) SARS-CoV-2 infection: unadjusted (n = 821)

Marker	N	−30 days	0 days	180 days	365 days	*P* value
HbA1c (%)	785	5.39 (0.01)	5.39 (0.01)	5.40 (0.01)	5.40 (0.02)	.435
Non-HDL cholesterol (mg/dL)	791	129.6 (1.2)	129.3 (1.2)	127.6 (1.2)	125.8 (1.4)	<.001
Troponin (ng/L)	139	7.31 (0.61)	7.29 (0.59)	7.17 (0.54)	7.05 (0.63)	.681
Pro-BNP (pg/mL)	756	68.5 (3.1)	68.6 (3.1)	68.2 (3.2)	69.7 (3.5)	.443
D-dimer (ng/mL)	768	0.73 (0.27)	0.76 (0.30)	0.94 (0.49)	1.12 (0.68)	.336
Hs-CRP (mg/L)	764	3.03 (0.17)	3.05 (0.17)	3.13 (0.20)	3.22 (0.26)	.318
Vitamin D (ng/mL)	777	92.9 (1.3)	93.0 (1.3)	93.6 (1.4)	94.2 (1.6)	.250
Cystatin C (mg/L)	740	0.87 (0.01)	0.87 (0.01)	0.89 (0.01)	0.91 (0.01)	<.001
BMI (Kg/m^2^)	818	27.2 (0.20)	27.2 (0.20)	27.2 (0.20)	27.3 (0.21)	.211
Systolic BP (mmHg)	820	123.4 (0.5)	123.3 (0.5)	122.9 (0.5)	122.4 (0.5)	<.001
Diastolic BP (mmHg)	820	76.6 (0.3)	76.6 (0.3)	76.4 (0.3)	76.3 (0.3)	.04
Waist circumference (cm)	817	92.2 (0.5)	92.2 (0.5)	92.2 (0.5)	92.3 (0.5)	.562

Values are presented as mean (SE).

Abbreviations: BMI, body mass index; BP, blood pressure; hs-CRP, high-sensitivity C-reactive protein; non-HDL, non-high-density lipoprotein; pro-BNP, pro-B-type natriuretic peptide.

Longitudinal trajectories of cardiometabolic biomarkers before and after SARS-CoV-2 infection accounting for LC status are shown in Table 3. Non-HDL cholesterol decreased over time in the overall cohort (β = −.0091 mg/dL/day; approximately −3.3 mg/dL over 1 year; *P* = .003). The hs-CRP (β = .0012 mg/L/day; ∼0.44 mg/L/year; *P* = .047), vitamin D (β = .0075 ng/mL/day; ∼2.7 ng/mL/year; *P* = .041), cystatin C (β = .0001 mg/L/day; ∼0.04 mg/L/year; *P* < .001), and BMI (β = .0006 kg/m^2^/day; ∼0.22 kg/m^2^/year; *P* = .042) increased over time, while systolic blood pressure decreased slightly (β = −.0024 mmHg/day; ∼−0.9 mmHg/year; *P* = .012). No significant time trends were observed for HbA1c, troponin, pro-BNP, D-dimer, diastolic blood pressure, or waist circumference.

**Table 3:** Trajectories of cardiovascular/metabolic markers before and after (up to 1 year) SARS-CoV-2 infection considering LC groups

Marker	N	β-time	*P* value time	β-LC1	*P* value LC1	β-LC2	*P* value LC2	β-LC1 × t	*P* value LC1 × t	β-LC2 × t	*P* value LC2 × t
HbA1c (%)	748	.00001	.878	.01	.721	−.03	.520	.00002	.813	.00001	.869
Non-HDL cholesterol (mg/dL)	751	−.0091	.003	1.48	.557	2.81	.499	−.005	.287	.01	.053
Troponin (ng/L)	132	.0011	.482	−.07	.942	−2.84	<.001	−.0006	.747	.00002	.991
Pro-BNP (pg/mL)	723	.0097	.101	3.23	.492	−4.75	.508	−.0098	.272	−.0199	.035
D-dimer (ng/mL)	734	.0002	.371	.75	.272	−.029	.380	.003	.256	.0003	.321
hs-CRP (mg/L)	729	.0012	.047	.69	.061	1.09	.072	−.0016	.140	.0001	.947
Vitamin D (ng/mL)	742	.0075	.041	−3.6	.201	−1.12	.803	−.005	.389	−.007	.575
Cystatin C (mg/L)	707	.0001	<.001	.035	.013	.019	.386	.00001	.816	.00001	.675
BMI (kg/m^2^)	775	.0006	.042	1.13	.008	1.84	.009	−.0007	.074	−.0007	.224
Systolic BP (mmHg)	777	−.0024	.012	.40	.705	−2.38	.097	−.0009	.518	.0019	.324
Diastolic BP (mmHg)	777	−.0006	.415	−.16	.805	.18	.848	−.001	.279	−.0003	.842
Waist circumference (inches)	774	.001	2.98	1.17	.005	4.59	.010	−.0009	.316	−.0031	.046

LC1: LC indeterminate (LCRI 1-10) vs LC− (LCRI = 0) reference group.

LC2: LC+ *(*LCRI ≥ 11) vs LC− (LCRI = 0) reference group.

Abbreviations: BMI, body mass index; BP, blood pressure; hs-CRP, high-sensitivity C-reactive protein; non-HDL, non-high-density lipoprotein; Pro-BNP, pro-B-type natriuretic peptide.

**Figure 2:**
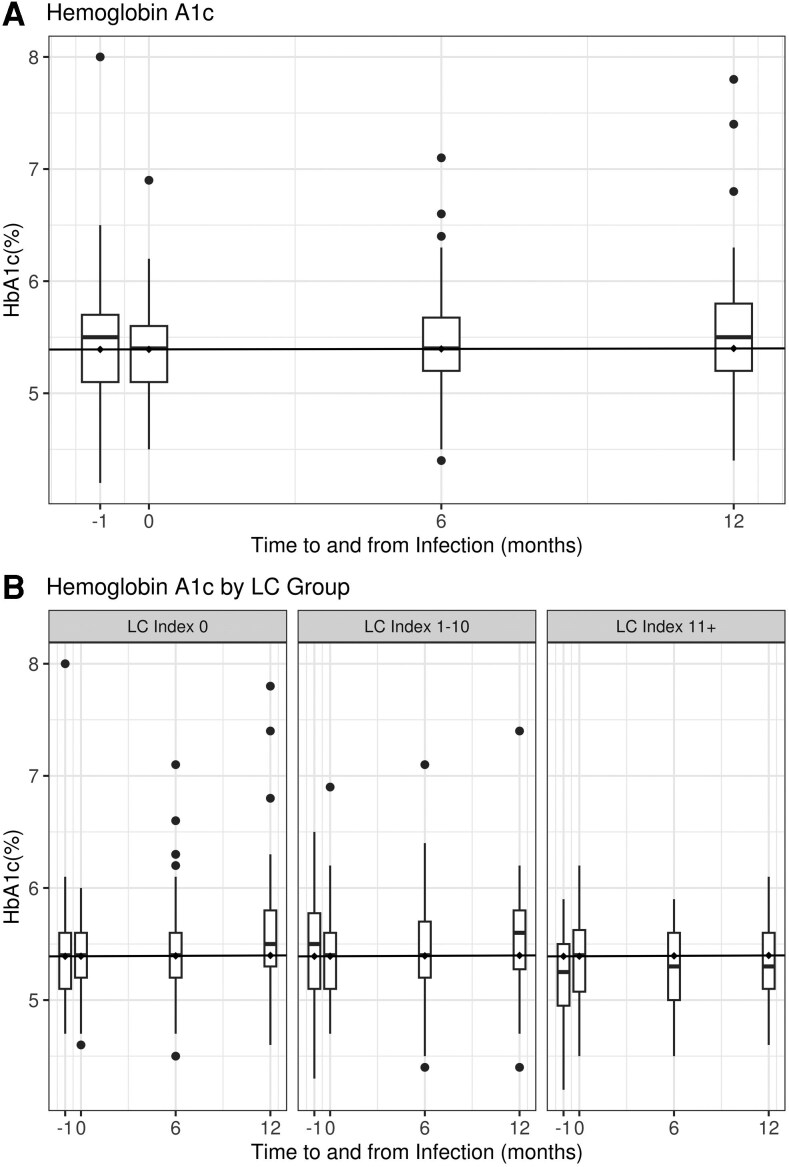
Longitudinal trajectories of HbA1c before and after SARS-CoV-2 infection. Panel (A) shows the distribution of HbA1c (%) in the overall cohort at 1 month pre-infection, acute infection, 6-month, and 12-month study visits. Panel (B) shows HbA1c distributions stratified by Long COVID (LC) status using the RECOVER Adult Long COVID Research Index (LCRI): LC− (LCRI = 0), LC indeterminate (LCRI 1-10), and LC+ (LCRI ≥ 11). Boxplots represent the median and interquartile range, with whiskers indicating values within 1.5 times the interquartile range; individual points represent outliers. The black line represents the mean HbA1c trajectory across time points. HbA1c remained stable from pre-infection through 12 months after infection, with no significant longitudinal change and no evident divergence across LC groups.

**Figure 3:**
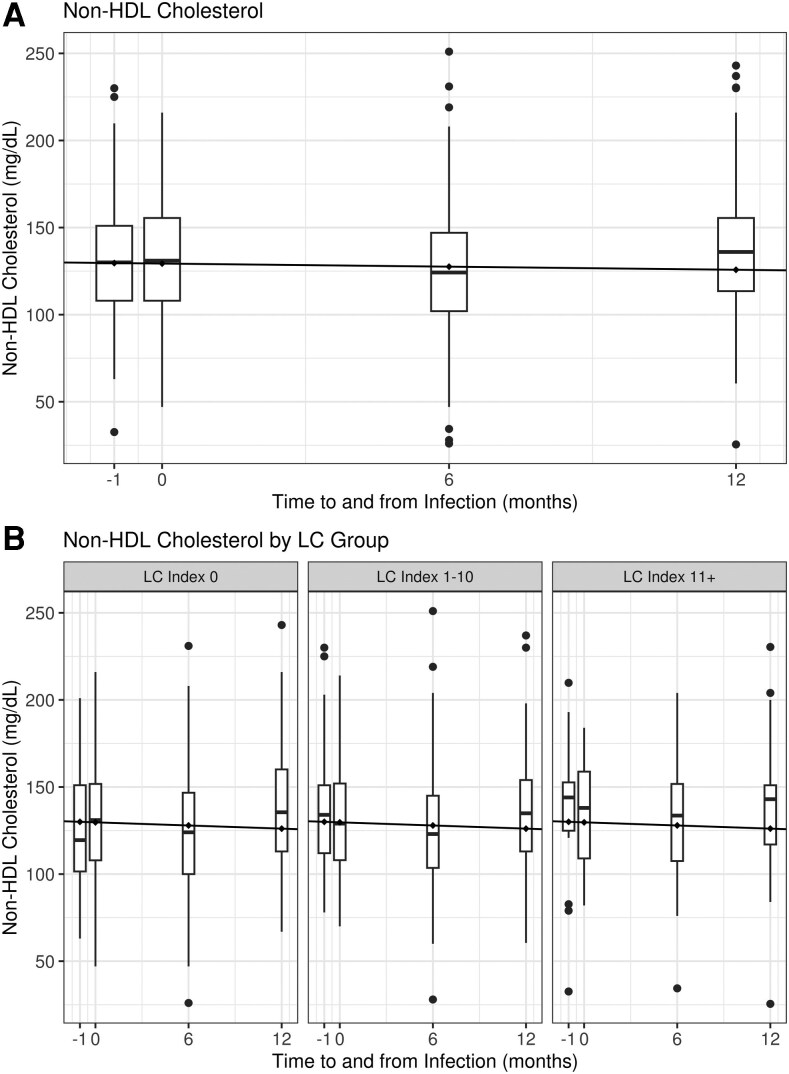
Longitudinal trajectories of non–HDL cholesterol and cystatin C before and after SARS-CoV-2 infection. Panel (A) shows the distribution of non-HDL cholesterol (mg/dL) in the overall cohort at 1 month pre-infection, acute infection, 6-month, and 12-month time points. Panel (B) shows non-HDL cholesterol distributions stratified by Long COVID status using the RECOVER Adult LCRI: LC− (LCRI = 0), LC indeterminate (LCRI 1-10), and LC+ (LCRI ≥ 11). Boxplots represent the median and interquartile range, with whiskers extending to values within 1.5 times the interquartile range; individual points represent outliers. The black line with points represents the mean non-HDL cholesterol trajectory across time points. Non-HDL cholesterol decreased modestly over follow-up in the overall cohort, with no clear consistent divergence across LC groups.

**Figure 4:**
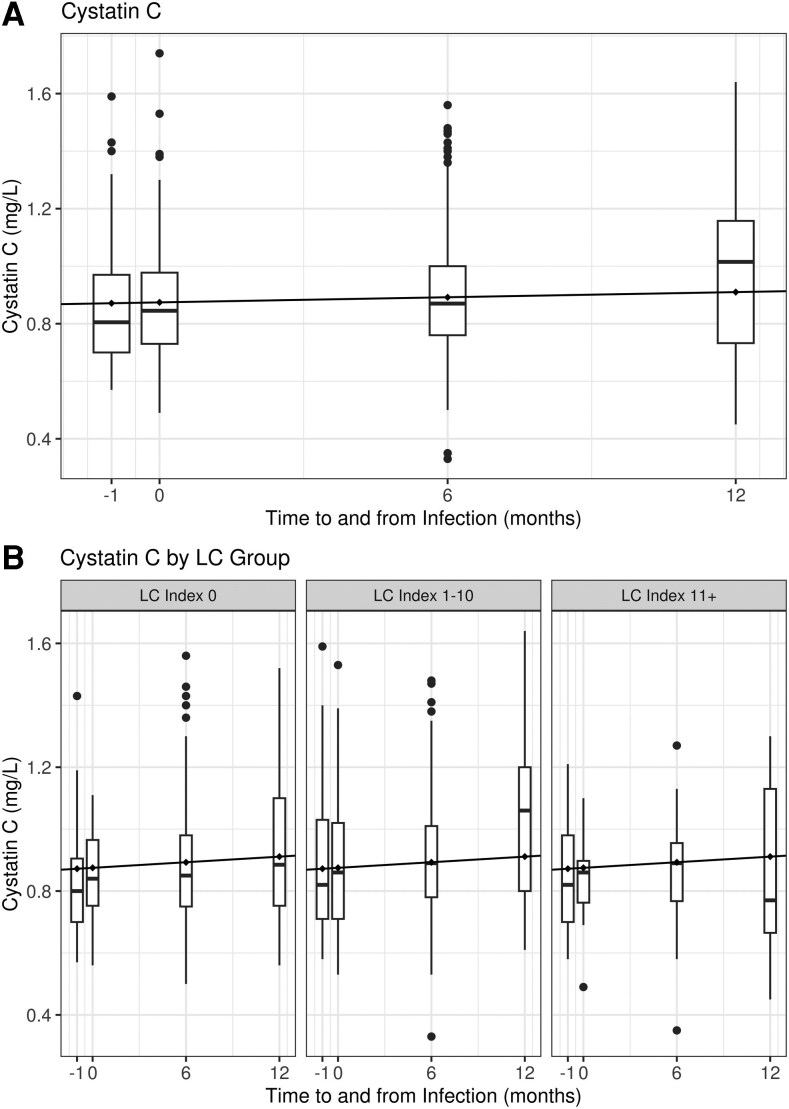
Longitudinal trajectories of non-HDL cholesterol and cystatin C before and after SARS-CoV-2 infection. Panel (A) shows the distribution of cystatin C (mg/L) in the overall cohort at 1 month pre-infection, acute infection, 6-month, and 12-month study visits. Panel (B) shows cystatin C distributions stratified by LC status the RECOVER Adult LCRI: LC− (LCRI = 0), LC indeterminate (LCRI 1-10), and LC+ (LCRI ≥ 11). Boxplots represent the median and interquartile range, with whiskers extending to values within 1.5 times the interquartile range; individual points represent outliers. The black line with points represents the mean cystatin C trajectory across time points. Cystatin C increased slightly over follow-up in the overall cohort, with no clear consistent divergence across LC groups.

Before COVID infection, subjects who were later classified in the LCI group had higher cystatin C (β = .035 mg/L; *P* = .013), BMI (β = 1.13 kg/m^2^; *P* = .008), and waist circumference (β = 1.17 inches; *P* = .005) compared with LC− participants. Before the infection, LC+ individuals had lower troponin levels (β = −2.84 ng/L; *P* < .001) and higher BMI (β = 1.84 kg/m^2^; *P* = .009) and waist circumference (β = 1.81 inches; *P* = .010). We did not observe any statistically significant longitudinal change in biomarkers when accounting for LC status except for pro-BNP that decreased in the LC+ group (β = −.0199 pg/mL/day; approximately −7.3 pg/mL/year; *P* = .035), when compared to LC− group. Visual representation of longitudinal changes of HbA1c, cystatin C, and non-HDL cholesterol are shown in Figs. 2-4, respectively.

### Changes in 10-year ASCVD and metabolic syndrome

As shown in Table 4, among 592 participants with available data, the estimated 10-year ASCVD risk increased from 6.7% before infection to 7.3% post-infection (*P* < .001). The 2 measurements were obtained on average 1.6 years apart. When stratified by LC status (Table 4), 10-year ASCVD risk increased modestly in all groups. The mean ASCVD risk increased from 6.5% to 6.9% (*P* = .005), from 7.0% to 7.6% (*P* < .001), and from 4.8% to 5.9% (*P* = .012) among the LC−, LCI, and LC+ groups respectively.

**Table 4:** ASCVD before and after COVID infection overall and by worst PASC score during follow-up

Groups	n	Age before infection	ASCVD before infection	ASCVD post-infection	Interval (years)	*P* value
Overall	592	52.3 ± 15.0	6.7% (± 12%)	7.3% (± 12.5%)	1.6 ± 0.6	<.001
LC−	244	53.1 ± 14.9	6.5% (±10.6%)	6.9% (± 11%)	1.5 ± 0.6	.005
LCI	261	52.6 ± 14.5	7.0% (±12.9%)	7.6% (± 13.4%)	1.6 ± 0.6	<.001
LC+	56	48.4 ± 15.6	4.8% (± 8%)	5.9% (± 9.7%)	1.7 ± 0.5	.012

Abbreviations: ASCVD, atherosclerotic cardiovascular disease; LC, long COVID, LCI, long COVID indeterminate; PASC, post-acute sequelae of SARS-CoV-2 infection.

The prevalence and incidence of metabolic syndrome before and after SARS-CoV-2 infection stratified by worst LC status are shown in Table 5. Among 815 participants, 483 (59.3%) did not meet metabolic syndrome criteria either before or after infection, while 151 (18.5%) developed new metabolic syndrome following infection. A total of 152 participants (18.7%) had metabolic syndrome both before and after infection, and 29 (3.5%) no longer met criteria after infection.

**Table 5:** Prevalence and incidence of metabolic syndrome before and after infection overall and by post-infection worst LCRI

Metabolic syndrome	Overall	LC−	LCI	LC+
N	815	339	354	82
No MetS before and after infection	483 (59.3%)	219 (64.6%)	202 (57.1%)	38 (46.3%)
New MetS post-infection	151 (18.5%)	65 (19.2%)	64 (18.1%)	16 (19.5%)
Persistent MetS pre- and post-infection	152 (18.7%)	48 (14.2%)	75 (21.1%)	23 (28.0%)
Recovered after infection	29 (3.5%)	7 (2.0%)	13 (3.7%)	5 (6.1%)

Abbreviations: LC, long COVID, LCI, long COVID indeterminate; MetS, metabolic syndrome.

When stratified by LC status, the proportion of participants without metabolic syndrome both before and after infection was highest in the LC− (64.6%), compared with 57.1% in the LCI group and 46.3% in the LC-positive group. The proportion of participants with metabolic syndrome both before and after infection increased across LC categories, from 14.2% in the LC− group to 21.1% in the LCI group and 28.0% in the LC+ group. The incidence of new metabolic syndrome after infection was similar across LC groups (19.2% in LC−, 18.1% in LCI, and 19.5% in LC+). Recovery from metabolic syndrome was observed in 2.0%, 3.7%, and 6.1% of participants in the LC−, LCI, and LC+ groups, respectively.

## Discussion

In this prospective cohort using available laboratory data prior to infection and up to 12-month post infection, we did not observe clinically meaningful deterioration in glycemic control, inflammatory and cardiometabolic biomarkers, with a significant but modest increase in the estimated 10-year ASCVD risk following SARS-CoV-2 infection. HbA1c measures remained stable throughout the study period with no evidence of increased incidence of post-infection new-onset diabetes. Similarly, troponin, pro-BNP, D-dimer, hs-CRP, BMI, and waist circumference did not show significant overall deterioration. Although several markers changed significantly over time, including non-HDL cholesterol, cystatin C, and blood pressure, the absolute magnitude of these differences was generally small, suggesting that statistical significance did not necessarily translate into clinically meaningful deterioration.

In our cohort, HbA1c remained stable across pre-infection, acute phase, 6 and 12 months post-infection in the total cohort, and across the 3 different LC groups. Our results contrast with other multiple large observational studies reporting increased incidence of newly diagnosed diabetes following COVID-19 (4, 6). Similarly, a metanalysis of 9 studies found increased risk of diabetes incidence post COVID-19 with relative risks ranging from 1.3 to 1.7 compared with noninfected controls (18). These findings differ from several large retrospective registry-based studies reporting increased incidence of diabetes after COVID-19. The majority of those analyses relied on electronic health records diagnostic codes, or post-infection clinical encounters but lacked pre-infection data or relied on contemporary controls with the possibility of COVID-19 infection that has not been tested, making them susceptible to surveillance bias and misclassification of preexisting diabetes as incident. By contrast, our present study incorporated pre-infection measurements and longitudinal follow-up within the same individuals, allowing direct assessment of glycemic trajectories over time and mitigating these biases. Additionally, the high vaccination rate in our cohort, including the LC+ group, may have attenuated the metabolic consequences of COVID-19 infection and could explain the overall stability of HbA1c and cardiometabolic markers observed.

Despite the overall stability of most biomarkers among our cohort, several statistically significant trends were observed. Non-HDL cholesterol (from 129.6 mg/dL before infection to 125.8 mg/dL at 12 months) and both systolic and diastolic blood pressure decreased modestly over time (≤1 mmHg), while cystatin C increased slightly (0.87 mg/dL before infection to 0.91 mg/dL at 12 months). The reduction in non-HDL cholesterol and blood pressure would signal a more favorable post-infection profile, whereas the slight rise in cystatin C may indicate a subtle shift in kidney-related or inflammatory biomarker status, but these are small changes in absolute terms and difficult to interpret as clinically meaningful renal, inflammatory, and cardiometabolic dysfunction. Our findings may reflect behavioral or clinical factors occurring after infection, such as lifestyle changes, medication adjustments, or due to frequent clinical and laboratory monitoring within the RECOVER study, rather than direct biological effects of acute or post-acute SARS-CoV-2 infection. When analyses accounted for LC status, additional modest changes were observed in hs-CRP, vitamin D, and blood pressure; however, after accounting for multiple comparisons, only changes in non-HDL cholesterol and cystatin C remained statistically relevant. Although, the absolute magnitude of these changes remained small. It is of note that the estimated 10-year ASCVD risk increased slightly overall, from 6.7% pre-infection 7.3% post-infection, and increased similarly across the 3 LC groups, and this difference was statistically significant. However, the 2 measurements were obtained approximately 1.6 years apart. Since age is a direct component of the pooled cohort equation, this small increase likely reflects the expected effect of aging over time rather than a major COVID-related worsening in cardiovascular risk profile.

Taken together, these results suggest that modest longitudinal biomarker fluctuations occurred over follow-up, but not in a pattern consistent with major sustained cardiometabolic injury. However, the absence of major abnormalities in routine biomarkers does not exclude more subtle physiological disturbances after COVID-19. Prior mechanistic studies of post-acute sequelae of SARS-CoV-2 infection (PASC) have identified impairments in endothelial function, autonomic regulation, and exercise capacity despite largely normal standard laboratory measurements (19-22). Oxidative lipid modification has also been implicated; elevated oxidized LDL levels associated with vascular dysfunction have been reported in LC (23, 24). Thus, conventional cardiometabolic biomarkers may be insufficiently sensitive to detect microvascular, mitochondrial, or metabolic alterations occurring in some individuals with LC. This might also raise the hypothesis that some of the associations between LC and adverse cardiometabolic profile may reflect preexisting host susceptibility rather than a purely post-infection process.

There was a heterogeneity in post-infection metabolic syndrome trajectories. Some participants developed metabolic syndrome after infection, and others remained classified as having metabolic syndrome, but a non-negligeable number no longer met the metabolic syndrome criteria. This bidirectional movement argues against a uniformly progressive post-COVID metabolic phenotype across the cohort. Although 18.5% of participants developed new metabolic syndrome after infection, this proportion was similar across LC categories, suggesting that incident post-infection metabolic syndrome was not associated with LC status and reinforcing the importance of longitudinal data including pre-infection status. These descriptive data do not establish a causal mechanism and indeed may reflect reverse causality; that is, individuals with metabolic syndrome are more prone to LC with more severe and persistent symptoms.

Our study has several notable strengths, including, most importantly, objective pre-infection measurements which allowed longitudinal within-person comparisons, and stratification by LC status. The design also incorporated repeated follow-up across clinically relevant time points extending to approximately 1 year, and the analysis considered both overall trajectories and models incorporating LC groups. Several limitations should also be acknowledged. First, although many markers were measured repeatedly, not all participants had complete data for all biomarkers, and some markers such as troponin were available in fewer individuals. Second, LC grouping was based on self-reported symptoms which might infer reporting bias, and it was based on maximum post-infection symptom score, which may not fully capture the dynamic and heterogenous nature of symptoms over time.

## Conclusion

In this prospective cohort with pre-infection baseline data, COVID-19 infection was not associated with clinically meaningful worsening in HbA1c, inflammatory, and cardiometabolic markers over approximately 1 year of follow-up. These findings underscore the value of longitudinal pre- and post-infection assessment in distinguishing small physiological changes from clinically meaningful chronic disease after COVID-19. Future larger studies should evaluate longer-term metabolic outcomes beyond 1 year after COVID-19 infection, particularly among individuals with prediabetes, or other baseline cardiometabolic risk factors who may be more susceptible to progression toward dysglycemia. In addition, further investigation is needed to better understand the potential influence of vaccination status and viral variants on long-term metabolic outcomes and LC manifestations.

## Data Availability

Original data generated and analyzed during this study are included in this publication.

## Abbreviations

ASCVD: atherosclerotic cardiovascular disease
BMI: body mass index
HbA1c: glycated hemoglobin
HDL: high-density lipoprotein
hs-CRP: high-sensitivity C-reactive protein
LC: long COVID
LCI: long COVID indeterminate
LCRI: 2024 RECOVER Adult Long COVID Research Index
LDL: low-density lipoprotein
pro-BNP: pro–brain natriuretic peptide
